# UK-based clinical testing programme for somatic and germline BRCA1/2, ATM and CDK12 mutations in prostate cancer: first results

**DOI:** 10.1136/bmjonc-2024-000592

**Published:** 2025-02-24

**Authors:** D Gareth Evans, George Burghel, Helene Schlecht, Ashwin Sachdeva, Andrew Hudson, Omi Parikh, Fiona Lalloo, Robert Bristow, Emma R Woodward

**Affiliations:** 1 Genomic Medicine, Manchester Academic Health Science Centre, Manchester, UK; 2 The Christie NHS Foundation Trust, Manchester, UK; 3 Clinical Oncology, Royal Preston Hospital, Preston, UK; 4 Genomic Medicine, North West Genomic Medicine Service Alliance, Manchester, UK; 5 The University of Manchester, Manchester, UK

**Keywords:** Prostate cancer

## Abstract

**Objective:**

Germline *BRCA2* pathogenic variants (PVs) are known to cause ~4% of prostate cancer, but other homologous repair genes, *BRCA1, ATM, PALB2* and Lynch syndrome genes are also involved. Our objective was to assess the contribution of germline and somatic gene variants to prostate cancer.

**Methods and analysis:**

We reviewed germline/tumour DNA testing from 450 localised or metastatic prostate cancer cases in NW England mainly from 2022 to 2024. ORs for additional genes used detection rates in controls from the BRIDGES study.

**Results:**

450 cases underwent *BRCA1/2* germline/somatic testing with 2 germline PVs in *BRCA1* (0.4%) and 27 in *BRCA2* (6.0%)—total 6.4% and 6/328 (1.8%) in *ATM*. There were 280 metastatic prostate cancer samples tested with 11 (4%) somatic *BRCA2* and 7 (2.7%) somatic *ATM* identified with 1 somatic *BRCA1*. Total PVs in *BRCA2* were 31/280 (11%), including germline and indeterminate. *CDK12* somatic PVs were found in 9/220 (4.1%), including 2 digenic with *BRCA2* and 2 which were biallelic.

**Conclusion:**

In this continuous clinical evaluation, *BRCA2* is the most frequently identified prostate cancer gene with over 10% involvement in metastatic disease. *BRCA2* and *CDK12* somatic PVs do not appear to be mutually exclusive. *BRCA1* does not appear to be a significant contributor to prostate cancer progression.

**WHAT IS ALREADY KNOWN ON THIS TOPIC**

- Germline and somatic *BRCA2* pathogenic variants are known to be an important factor in prostate cancer and in particular in aggressive metastatic disease but the importance of *ATM, BRCA1* and *CDK4* is less well delineated.

**WHAT THIS STUDY ADDS**

- We show that germline or somatic *BRCA1* variants are not a major contributor to prostate cancer, although both germline and somatic *ATM* and *CDK4* are and show that *BRCA2* and somatic *CDK4* are not mutually exclusive.

**HOW THIS STUDY MIGHT AFFECT RESEARCH, PRACTICE OR POLICY**

- The low involvement of *BRCA1* may mean that tumours occurring in the context of a germline *BRCA*1 variant may not be driven by homologous repair deficiency and may not respond to poly(ADP-ribose) polymerase-1/2 inhibitors treatment.

## Background

 Prostate cancer is the most common male cancer and is heterogeneous in clinical presentation due to differential genomic and microenvironmental factors.^1^ Significant advancements in our understanding of the genetic basis of prostate cancer subtypes and randomised trials have supported the use of genetic variants in DNA damage repair and response (DDR) genes as predictive assays for targeted therapy. Men with *BRCA2-*mutated or *ATM*-mutated prostate cancer have an increased risk of developing aggressive disease and show relatively poor prognosis.^2^,^5^

Poly(ADP-ribose) polymerase-1/2 inhibitors (PARPi) are the first class of targeted therapies to show clinical benefit in prostate cancer and are used as standard therapy for genetically stratified metastatic and castrate-resistant prostate cancers (mCRPC) with BRCA-mutant (germline (g*BRCA*) or somatic (s*BRCA*)) or altered *PALB2* status.^6^,^8^ *ATM*-mutant metastatic prostate cancer is currently being assessed for response to ataxia telangiectasia and rad3-related inhibitor therapies.^9^ Additionally, *CDK12* biallelic loss is associated with focal tandem duplications^8 10^ and gene fusions, which create neoantigens and a strong immune response leading to benefit from immune checkpoint inhibitors in patients with *CDK12* deficiency.^11 12^

Somatic testing for s*BRCA2*, s*ATM* and s*CDK12* is now routine in mCRPC, in addition to National Health Service (NHS) germline testing in high-risk individuals in NHS secondary and tertiary cancer care settings.^13^,^15^ The experience of prostate cancer germline and somatic testing in the UK North-West Genomic Laboratory Hub (NWGLH) was assessed.

## Methods and analysis

Men diagnosed with prostate cancer, with a family history or aggressive clinical features, have undergone germline testing for *BRCA1/2* in the UK from September 1996 to March 2024. More recently, tumour s*BRCA1/2,* s*ATM* and s*CDK12* testing (January 2021–March 2024) using clinically validated assays in the NWGLH has been undertaken.^16^ Additional panel testing for prostate cancer index cases fulfilling criteria has taken place for *PALB2, ATM, MLH1, MSH2, MSH6* and *CHEK2* since April 2023 as a part of NHS Genomics England’s panel, R430. The Manchester Scoring System (MSS) was used to assess the likelihood of g*BRCA1/2* (online supplemental table 1).^17^

The timelines for each panel are recorded as follows.

1. 1996–2013: germline *BRCA1/2* testing based on a 20% threshold for identifying a *BRCA1/2* pathogenic variant (PV) MSS≥20.
2. 2013–2018: germline *BRCA1/2* testing based on a 10% threshold MSS≥15.
3. 2018–2024: germline *BRCA1/2* and *PALB2* testing based on a 10% threshold MSS≥15 with *ATM+CHEK2+RAD51C/D* in 2021.
4. 2023–2024: R430 germline testing of men with prostate cancer<50 years, diagnosed with metastatic prostate cancer<60 years or with prostate cancer with a family history of prostate cancer where estimated likelihood of identifying a PV is≥10% or Ashkenazi Jewish ancestry and any prostate cancer.

The next generation sequencing (NGS) tumour *BRCA1/2* assay has been previously reported.^16 18^ In brief, tumour DNA was extracted from formalin-fixed, paraffin-embedded blocks that contained ideally≥20% tumour content. These were nearly all from the primary prostate cancer biopsies. Bioinformatic analysis used an in-house pipeline validated to identify s*BRCA1/2,* s*ATM* and s*CDK12* at variant allele frequencies (VAFs)≥4%. The NGS assay detects single-nucleotide variants and small duplications, deletions and/or insertions≤40 base pairs across the whole coding sequence of *BRCA1*/*2*±15 base pairs beyond exon–intron junctions. Germline *BRCA1/2* and extended panel testing were performed on DNA extracted from peripheral circulating lymphocytes. The NGS and multiplex ligation-dependent probe amplification assays used to detect g*BRCA* PVs have also been previously reported.^16 18 19^ Variants detected on somatic analysis for which germline data were unavailable were considered somatic if the VAF was<34% with 0/20 with s*BRCA1/2* confirmed on blood from previous work.^18 19^ Similarly using≥67% VaF additional work from this study showed that all with≥67% VAF whose PV was seen in ≥10 families of n=1000 g*BRCA1* or n=1000 g*BRCA2* families in Manchester was considered germline as 0/20 were somatic using samples from this analysis as well as previous work.^18 19^ Samples without s*BRCA1,* s*BRCA2* and s*ATM* were considered to be negative for germline variants as concordance rate is extremely high with no variants missed apart from copy number variants (CNVs).^18^ Currently, only 4/142 (2.8%) germline variants were missed on dual somatic testing, all CNVs.

Variant interpretation was performed as per American College of Medical Genetics/American Molecular Pathology and UK guidelines^19 20^ ^21^ as per previously published papers.^16 18 19^ Briefly, variants that truncate the protein product and are before the last exon or last recorded PV, such as frameshift or non-sense variants, are typically in the studied genes and always considered PVs. Additionally, the great majority of canonical splicing variants are also usually considered at least likely pathogenic. Missense variants or in frame deletions, including whole exon, need additional supportive data, such as supportive case control studies.

The data included in this study were collected as a part of continuous clinical service evaluation. Clinical data were acquired at the time of reporting the germline and/or s*BRCA1/2* status. Statistical tests used Fisher exact test (two sided). Controls were taken from the BRIDGES study of 50 703 women from Western Europe with a high proportion from the UK.^22^ Briefly, each breast, ovarian, prostate and pancreas in the highest scoring lineage is scored with an age weighting. A combined adjusted score of 15–19 is equivalent to a 10% threshold rate for detecting a *BRCA1/2* PV.^17^ We also used our regional g*BRCA1* and g*BRCA2* database to assess the numbers of prostate cancer in families. Patients were not involved in the research.

## Results

### Population

**Figure 1:**
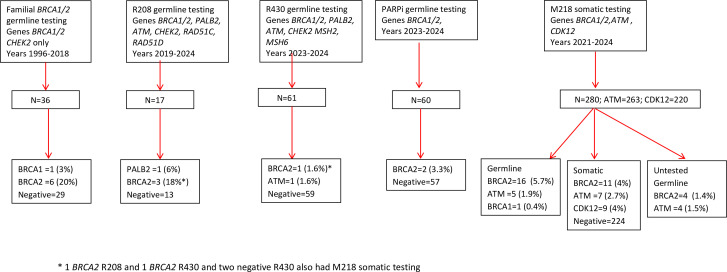
Flowchart on genetic testing for prostate cancer.

A total of 450 prostate cancer patients have received either germline (n=166), tumour somatic (n=280) or full gene testing of both (n=4) (tables1  2; figure 1).

**Table 1:** Germline testing of prostate, including inferred from somatic testing

	Number tested	Inferred from somatic only	PV	%	Possible PV from somatic	PC<60	PV		PC≥60	PV	%
*BRCA1*	450	275	2	0.44%	0	139	0	0.00%	311	2	0.64%
*BRCA2*	450	275	27	6.00%	4	139	13	9.35%	311	14	4.50%
*ATM*	328	257	6	1.83%	1	98	1	1.02%	230	5	2.17%
*PALB2*	97	0	1	1.03%	NA	75	0	0.00%	22	1	4.55%
*CHEK2*	122	0	0	0.00%	NA	87	0	0.00%	35	0	0.00%
*Lynch*	69	0	0	0.00%	NA	61	0	0.00%	8	0	0.00%
*RAD51C/D*	15	0	0	0.00%	NA	1	0	0.00%	14	0	0.00%

PCprostate cancerPSAprostate specific antigenPVPathogenic VariantPVpathogenic variant

**Table 2:** Tumour testing results

Result	Number tested	PV (inferred)	%PV	VAF range	Median VAF	PV absent from *BRCA2* germline database	%	P versus g*BRCA2*	Age range	Median	P versus g*BRCA2*
g*BRCA2*	280	16 (7)	5.71%	34%–89%	63%	4	25%	ref	50–79	59	ref
s*BRCA2*	280	11 (8)	3.93%	5%–69%	32%	9	81.82%	0.006	57–81	71	P<0.001
Uncertain s/g*BRCA2*	280	4	1.43%	39%–63%					50–83	81	
g*ATM*	262	5 (0)	1.91%	49%–63%	60%				65–74	71	P<0.001
s*ATM*	262	7 (5)	2.67%	6%–37%	26%				66–87	73	P<0.001
Uncertain *ATM*	262	4	1.53%	37%–60%					66–80	74	
s*CDK12*	220	9 (9)	4.09%	21%–44%	27%				50–81	71	P<0.001
g*BRCA1*	280	1 (0)	0.36%	48%					73		
s*BRCA1*	280	1 (1)	0.36%	5%	5%				61		
Negative	280	224	80.00%								

PVpathogenic variantVAFvariant allele frequency

The 340 patients submitted to either M218 somatic testing or germline PARPi testing with metastatic disease were aged 38–87 years (mean 69.1, median age 70.05 and IQR 64–75 years). Overall, of this metastatic population unselected for age or family history, at least 18/340 (5.3%) had a g*BRCA2* (figure 1) with only one g*BRCA1* (0.29%) and 7/263 (2.7%) g*ATM*. For the 124 undergoing germline testing based on age or family history, they were 34–77 years (mean 56.6, median age 56 and IQR 50–62 years). Four patients were in both cohorts. Again, g*BRCA2* predominated with 8/124 (8%) with only one (0.8%) g*BRCA1*.

Including inferred testing from tumours, effective germline results for *BRCA1/2* were available on all 450. There were 27 g*BRCA2* PVs (6.0%), compared with only 2 g*BRCA1* (0.44%). There were 6/328 (1.8%) g*ATM* PVs, but 1 was present in one of the g*BRCA1* heterozygotes. Otherwise, only a single g*PALB2* PV (in a man in his 70s with a strong breast cancer family history) (Manchester score=26) was identified in 97 with none in *CHEK2* or those undergoing Lynch (n=69) or *RAD51C/D* (n=15) testing.

In total, 280 patients with metastatic prostate cancer had successful M218 somatic testing (table 2). Overall, 31 (11.1%) had an identifiable PV in *BRCA2*. 16 (5.7%) were confirmed germline (9 from PV confirmatory blood testing and 7 inferred) and 11 (4%) were confirmed somatic (3 from blood and 8 inferred). Four samples were classified as being uncertain with the PV absent from the 1085 g*BRCA2* family database and VAFs of 39%, 43% and 64% in three and a further having a common g*BRCA2* PV with VAF=61%. 12/16 (75%) g*BRCA2* had suggestive data for biallelic inactivation with 11 having VAF>60% and a further case has a presumed germline PV at 49% VAF and a second single-nucleotide PV at 15%. Only 4/16 germline *BRCA2* PV was absent from the family database compared with 9/11 (83%) of somatic (p=0.006). The germline cases were younger than the somatic group (table 2). As the two lower VAFs (39% and 43%) in those with unconfirmed germline data were aged 81 and 83 and their variant was absent from the family database, these two were likely somatic. There was only one s*BRCA1* with VAF of 6%.

Overall, 16/263 (6.1%) tumour samples had an *ATM* PV identified. Five (1.8%) were germline (all confirmed on blood), seven (2.5%) somatic (two on blood and five inferred) and four indeterminate. One germline was biallelic with variants with VAF of 58% and 18% and another had a VAF of 83% strongly supporting the loss of wild type. VAF of the four indeterminate cases was 37%–60%.

There were 9 of 220 (4.1%) samples that showed *CDK12* PV. Two of these had biallelic PV with similar low VAF. Interestingly, two of the *CDK12* samples also had *BRCA2* PV. One had a germline PV with a VAF of 89% (*CDK12* VAF=26%) and another was somatic with the *BRCA2* VAF of 13% compared with *CDK12* of 20%. In total, 51/277 (18.5%) metastatic prostate samples had an actionable variant on somatic NGS.

There was a fairly high failure rate with only 217/478 (45.4%) tumour samples in the recent audit period passing for all genes tested. 104 samples failed due to inadequate material for analysis and a further 157 due to insufficient quantity/quality of DNA extracted. Of these fails, 42 had subsequent germline PARPi testing for *BRCA1/2* and 2 *BRCA2* PVs were found (figure 1).

Of the metastatic cases, 33/338 (9.9%) (M218 and PARPi panels) had an actionable s*BRCA2* PV with at least 18 (5.6%) being germline. The detection rate was as high, including somatic, as for germline PV in familial selected samples 10/100 (10.0%), although four samples were in each dataset (figure 1). The OR using the BRIDGES control frequencies^14^ for g*BRCA2* and g*ATM* was 21.11 and 6.03, respectively. Given the coexistent g*ATM* in the only g*BRCA1* cases, ORs were not carried out.

The Manchester score, where family history was available, was predictive as increasing detection rate was associated with an increasing Manchester score (table 3). However, the great majority of the 322 metastatic samples did not have family history supplied. Nonetheless, g*BRCA2* detection rates were low in apparently sporadic metastatic prostate≥60 (2.1%). Of familial prostate samples with no family history of breast, ovary or pancreatic cancer, 0/33 with a Manchester score for *BRCA2* of ≥4 equivalent to two prostate cancers<60 or one<60 and two over 60 had a g*BRCA*. This includes 0/11 with a score for g*BRCA2* of≥6 equivalent to three prostate cancers<60.

**Table 3:** Detection rate of *BRCA1/2* combined by Manchester score

MSS	Apparently isolated PC≥60	Apparently isolated PC<60	3–14	15–20	20+
Number tested	236	116	69	13	16
*BRCA1/2* PV	5	8	7	3	6
%	2.12%	6.90%	10.14%	23.08%	37.50%

MSSManchester scoring systemPVpathogenic variant

### g*BRCA1/2* register data

A genetic register for our families, including cascading testing and a dedicated database, has been available since 1996.^20 23^ There are currently 1054 g*BRCA1* and 1084 g*BRCA2* families included. Within these families, there were 19/435 (4.4%) male g*BRCA1* heterozygotes with prostate cancer of whom 7 (37%) had died. In contrast, there were 66/507 (13%) male g*BRCA2* heterozygotes with prostate cancer (p<0.0001) of whom 43 (65%) had died (p=0.03). 34 (51.5%) of the g*BRCA2* prostate cancers had died within 5 years of diagnosis compared with only 4 (21%) for g*BRCA1*(p=0.02). All eight prostate cancers (g*BRCA2*=5) diagnosed in the IMPACT PSA screening trial (not a part of the testing in the tables as presymptomatic) are alive without metastatic disease in follow-up as long as 17 years.^24^

## Discussion

The current study within a busy clinical urogenital service supports the major involvement of *BRCA2* in metastatic prostate cancer with a combined s*BRCA2* and g*BRCA2* rate of at least 9.8%. This is in contrast to *BRCA1* with only one somatic variant and one germline PV in metastatic disease (1/338) found in combination with a g*ATM* PV. Given the far more convincing association of prostate cancer with both somatic and g*ATM* PVs in metastatic disease among 18/263 (6.8% germline 1.9%), even this 1 *BRCA1* PV appears unlikely to be a driver in that tumour. In their 2022 paper, Abdi *et al* found the germline rates of 4.1%, 2% and 0.5% for *BRCA2, ATM* and *BRCA1* in 557 patients with metastatic disease; these are similar to our rates of 5.7%, 1.9% and 0.3% in 322 cases. Combining with the eight studies reported by Abdi *et al,*^13^ there were 179 (3.84%) germline *BRCA2,* 68 (1.46%) germline *ATM* and only 27 (0.58%) germline *BRCA1* in 4664 men with metastatic prostate cancer. Although population frequencies of g*BRCA1* vary from 0.11% to 1.1%,^22 25^ there are no control rates reported.^13^ In contrast, population rates for *BRCA2* of 0.27%–1.5%^22 25^ are clearly much lower than in metastatic disease as well as for *ATM* (0.3%).^22^ The upper rates for *BRCA1/2* are really only seen in the Ashkenazi Jewish population.^25^

Original studies suggested that g*BRCA1* was associated with an increased risk of early onset prostate cancer with an OR of 3.3 found in 33 families linked to *BRCA1*.^26^ However, more recent larger studies have called into question this link with ORs of 0.82 (95% CI 0.54 to 1.27) in 3154 families^27^ and 1.0 (95% CI 0.4% to 2.3%) in 268 *BRCA1* families.^28^ We have also shown a threefold higher prevalence of prostate cancer among *BRCA2* heterozygotes with the expected higher death rates. Even if there is a signal for non-metastatic prostate cancer in *BRCA1,* this may not justify PSA screening, given the high rates of overdiagnosis. It may be time, therefore, to question whether*BRCA1* should be considered to be a prostate cancer predisposing gene, given its very low prevalence in the present study of somatic mutations. However, for BRCA2, there appears to be some support for PSA screening from the IMPACT study,^24^ particularly in view of the known higher mortality rates.^2^,^513 29^

The results of somatic testing are interesting from the current study. To assess their significance, we accessed a recent systematic review.^30^ However, this review has some substantial flaws. A high proportion of the studies sampled as ‘somatic’ did not have verification of germline status. This includes the use of the Cancer Genome Atlas 2015^31^a Japanese^32^ and Belgian study.^33^ The review assigned all *BRCA1/2* PVs found on liquid biopsy on ctDNA to the somatic group even though most were predicted by the authors to be germline.^34^ Most worryingly, the founder Jewish *BRCA1/2* PVs identified on testing of tumours in an Israeli study were all assigned to the somatic group despite these being almost certain to be germline.^35^ Confining the analysis to series in which tumour sequencing had been performed on metastatic prostate cancer^36^,^39^ and definitive PVs had been identified within the four genes assessed in the present study (including homozygous deletion), there were a total of 694 patients found including the current study (table 4). There was only one s*BRCA1* variant, with s*BRCA2* PVs being the most frequent at 5.0%, s*CDK12* (3.3%) and *ATM* (2.7%).

**Table 4:** Tumour sequencing studies of metastatic prostate cancer and detection rates of somatic (non-germline) variants in *BRCA1, BRCA2, ATM* and *CDK12*

Study	Number assessed	*ATM*	%	*BRCA1*	%	BRCA2	%	*CDK12*	%
Robinson *et al*^39^	150	6	4.00%	0	0.00%	11	7.33%	NA	
Mateo *et al*^38^	50	2	4.00%	0	0.00%	4	8.00%	NA	
Mota *et al*^37^	140	2	1.43%	0	0.00%	8	5.71%	4	2.86%
Jiang *et al*^36^	74	1	1.35%	0	0.00%	1	1.35%	3	4.05%
Present study	280	7/263	2.50%	1	0.36%	11	3.92%	9/220	4.1%
Total	694	18/677	2.66%	1	0.14%	35	5.04%	16/481	3.32%

A much larger recent study combining data from four multicentre observational studies analysed tumour and germline on 729 cases of metastatic disease.^3^ They reported 19 cases with *BRCA1* involvement, including 6 (0.8%) germline. However, they included deletions of the entire gene as involvement. Given that 17q loss is one of the most common events in cancer, it is not clear from the article whether any of the ‘somatic’ cases represents a true targeted *BRCA1* abnormality. This is further supported by the very low biallelic variant rate of 16% compared with 65% for *BRCA2*, similar to that identified in the current study. Given the inclusion of hemizygous deletion that may not target the specific gene, we were not able to add the data from this large study to table 4. In the absence of clear data from this article,^3^ the true rate of somatic *BRCA1* PVs appears extremely low. As g*BRCA1* PVs affect ~1 in 400–800, it is not surprising that we picked up a single g*BRCA1* as a potential chance finding.

We have not so far identified a g*MSH2/*g*MSH6* through R430 panel testing (yet). However, the numbers are still small (n=67). For context compared with g*BRCA1,* 14/308 (4.5%) male heterozygote g*MSH2* and 4/176 (2.3%) of g*MSH6* have been diagnosed with prostate cancer in our region, but these are younger than the g*BRCA1* series.

In their recent review, Cussenot *et al*^7^ noted that *BRCA2* and *CDK12* involvement were mutually exclusive. Our data suggest differently. We identified 2 of 220 with involvement of both genes. Given one of these had a *BRCA2* germline variant with very clear evidence of biallelic loss of function with a VAF of 89% and the other had somatic PVs in both genes, this seems unlikely to be coincidental. This may mean that these patients could benefit from either PARPi, immunotherapy or both. Biallelic *CDK12* involvement was also common in our study. No studies that we could find have reported that germline *CDK12* impacts prostate cancer risk. We did not find any evidence of g*CDK12* in the 220 tumours analysed.

We found a single g*PALB2* PV in 97 cases (1%). The potential link with prostate cancer is unclear. The largest cohort study of 524 families found no increased risk ‘The prostate cancer RR was estimated to be 0.42 (95% CI 0.21 to 0.84; p=0.014).^40^ However, a more recent review did suggest some link with aggressive prostate cancer.^41^ Further research is required to assess the utility of testing for *PALB2* in prostate cancer. Although Manchester score works well in the context of breast and ovarian cancer, it is likely to underestimate risk in young men with high Gleason score metastatic disease. Modifications are now being considered.

The current study has some limitations. We were not able to classify all PVs as either somatic or germline. Nonetheless, our ability to differentiate germline and somatic from tumour-only analysis appears robust.^19^ This is important as ‘tumour first’ is an increasingly common approach with the increasing provision of genomics in the mainstream setting. There is some loss of sensitivity for germline CNVs on somatic analysis but we predicted that this will have missed no more than 3% of germline variants from tumour-only cases. There was also a high failure rate on somatic testing, but we do not predict that this will affect the proportions with PVs identified. Furthermore, the study was not a systematic screening of all men presenting with prostate cancer at diagnosis (eg, aggressive localised or metastatic disease), so the case indices for de novo hormone-sensitive metastatic or localised prostate cancer cannot be calculated with confidence. These data will be important in determining the utility of DDR inhibitors in combination with androgen receptor signalling inhibitors in earlier stage localised or hormone-sensitive disease, compared with upfront taxane therapy, as a means to improve outcomes in these men who have adverse outcomes. As the population tested is relatively newly diagnosed with most diagnostic testing (>80%) in the last 2 years implications on outcomes, metastatic disease cannot be confidently asserted for g*BRCA1*. However, historical data from our genetic register do suggest better prognosis in g*BRCA1* compared with g*BRCA2*. We were not able to provide Gleason score for a large proportion of our cohort, but the association of g*BRCA2* with high Gleason score is well established.^2 4 42 43^ The poorer survival in g*BRCA2* is also well described.^2 4 43^

The strengths of the study include the largest we could find with reporting that allowed distinction between somatic and germline without the inclusion of hemizygous deletions, as evidenced in table 4.

## Conclusion

The current study has shown a high frequency of *BRCA2* involvement in both metastatic and familial prostate cancer of ~10%, including somatic PVs. In contrast, *BRCA1* is not convincing for either somatic or germline involvement in metastatic disease in our series. *Both* germline and somatic *ATM* mutations are also associated with metastatic disease. Our data suggest that s*CDK12* and s/g*BRCA2* should no longer be considered mutually exclusive.

## supplementary material

10.1136/bmjonc-2024-000592 online supplemental file 1

## Data Availability

Data are available on reasonable request.
