# Supplementary material for: UK-based clinical testing programme for somatic and germline BRCA1/2, ATM and CDK12 mutations in prostate cancer: first results
Source: BMJ Oncol. 2025 Feb 24;4(1):e000592. doi: 10.1136/bmjonc-2024-000592 (PMC11880781; doi:10.1136/bmjonc-2024-000592)
Supplement: online supplemental file 1 [file bmjonc-4-1-s001.pdf]

Supplementary table 1: Pathology adjusted Manchester Score criteria

Cancer and age (in years) at diagnosis -Score each cancer in a direct lineage in the family add BRCA1 & BRCA2 for combined score	*BRCA1*	*BRCA2*
FBC <30	6	5
FBC 30-39	4	4
FBC 40-49	3	3
FBC 50-59	2	2
FBC >59	1	1
MBC <60	5	8
MBC >60	5	5
Ovary <60	8	5
Ovary >60	5	5
Pancreas	0	1
Prostate <60	0	2
Prostate >60	0	1

Pathology adjustment

**Breast cancer**	**BRCA1 adjustment**	**BRCA2 adjustment**
HER2 positive	-6 **+/- grade ER**	0
Lobular	-2 +/- ER status	0
DCIS only	-2 +/- ER status	0
LCIS only	-4 **no other adjustment**	0
Grade 1 IDC	-2 +/- ER status	0
Grade 2 IDC	0 +/- ER status	0
Grade 3 IDC	+2 +/- ER status	0
ER positive	-1 +/- grade	0
ER negative	+1 +/- grade	0

Triple negative	+4 +/- grade i.e. grade 3 TNT= +6	0
**Ovarian cancer**		
High grade serous <60	+2	0
Epithelial (endometrioid, serous, clear cell) granulosa cell	none	none
Mucinous, borderline or germ cell tumours (except granulosa cell)	no score	no score
**Adopted**		
no known status in birth family	+2	+2

FBC = Female Breast Cancer
MBC = Male Breast Cancer
Pancreas either gender
